# Supplementary material for: Effects of Dietary Lysine Levels on Growth Performance, Nutrient Digestibility, Serum Metabolites, and Meat Quality of Baqing Pigs
Source: Animals (Basel). 2022 Jul 23;12(15):1884. doi: 10.3390/ani12151884 (PMC9330884; doi:10.3390/ani12151884)
Supplement: Supplementary file 1 [file animals-12-01884-s001.zip › animals-1735896-supplementary.pdf]

Table S1. Analyzed composition of diets (%).

Item	20-40 kg					40-60 kg					60-90 kg				
	1	2	3	4	5	1	2	3	4	5	1	2	3	4	5
Dry Matter	89.2	89.2	88.8	89.2	88.6	89.1	89.1	89.4	89.5	89.5	88.2	88.2	87.8	88.0	88.1
Protein (N × 6.25)	16.9	16.2	16.3	16.5	6.4	14.6	14.6	14.7	14.8	14.8	13.6	13.4	13.4	13.1	13.2
Crude fiber	3.85	4.35	3.94	4.14	4.50	5.21	5.93	6.54	6.57	6.44	7.17	7.66	7.69	7.77	7.98
Ash	4.21	4.20	4.22	4.40	4.30	4.17	4.04	4.07	4.23	4.13	4.44	4.54	4.32	4.44	4.36
Ether extract	2.03	2.09	2.05	1.97	2.08	2.39	2.17	2.24	2.18	2.23	2.18	2.04	2.15	2.07	2.25
Gross energy (Kcal/kg)	3950	3957	3905	3960	3938	3960	3965	3987	3984	3994	3927	3910	3893	3906	3889
Lysine	0.75	0.87	1.00	1.11	1.23	0.60	0.72	0.84	0.92	1.02	0.52	0.62	0.74	0.83	0.93
Methionine	0.17	0.15	0.16	0.18	0.16	0.12	0.12	0.14	0.13	0.14	0.13	0.15	0.13	0.14	0.13
Met+Cys	0.49	0.49	0.50	0.51	0.49	0.42	0.41	0.44	0.43	0.44	0.44	0.43	0.38	0.42	0.41
Threonine	0.81	0.80	0.81	0.81	0.80	0.70	0.68	0.67	0.67	0.65	0.64	0.62	0.57	0.61	0.61
Isoleucine	0.60	0.59	0.61	0.59	0.60	0.53	0.53	0.54	0.54	0.54	0.46	0.47	0.47	0.44	0.46
Valine	0.67	0.64	0.66	0.65	0.66	0.58	0.59	0.58	0.59	0.59	0.52	0.53	0.52	0.51	0.52
Leucine	1.56	1.54	1.57	1.54	1.53	1.53	1.53	1.55	1.51	1.53	1.58	1.53	1.42	1.53	1.45
